# The p53 response in single cells is linearly correlated to the number of DNA breaks without a distinct threshold

**DOI:** 10.1186/1741-7007-11-114

**Published:** 2013-11-19

**Authors:** Alexander Loewer, Ketki Karanam, Caroline Mock, Galit Lahav

**Affiliations:** 1 Department of Systems Biology, Harvard Medical School, Boston, MA 02115, USA; 2 Berlin Institute for Medical Systems Biology, Max Delbrueck Center for Molecular Medicine, 13125 Berlin-Buch, Germany

**Keywords:** Double strand breaks, p53, Live imaging, Single cells, Pulses

## Abstract

**Background:**

The tumor suppressor protein p53 is activated by cellular stress. DNA double strand breaks (DSBs) induce the activation of the kinase ATM, which stabilizes p53 and activates its transcriptional activity. Single cell analysis revealed that DSBs induced by gamma irradiation trigger p53 accumulation in a series of pulses that vary in number from cell to cell. Higher levels of irradiation increase the number of p53 pulses suggesting that they arise from periodic examination of the damage by ATM. If damage persists, additional pulses of p53 are triggered. The threshold of damage required for activating a p53 pulse is unclear. Previous studies that averaged the response across cell populations suggested that one or two DNA breaks are sufficient for activating ATM and p53. However, it is possible that by averaging over a population of cells important features of the dependency between DNA breaks and p53 dynamics are missed.

**Results:**

Using fluorescent reporters we developed a system for following in individual cells the number of DSBs, the kinetics of repair and the p53 response. We found a large variation in the initial number of DSBs and the rate of repair between individual cells. Cells with higher number of DSBs had higher probability of showing a p53 pulse. However, there was no distinct threshold number of breaks for inducing a p53 pulse. We present evidence that the decision to activate p53 given a specific number of breaks is not entirely stochastic, but instead is influenced by both cell-intrinsic factors and previous exposure to DNA damage. We also show that the natural variations in the initial amount of p53, rate of DSB repair and cell cycle phase do not affect the probability of activating p53 in response to DNA damage.

**Conclusions:**

The use of fluorescent reporters to quantify DNA damage and p53 levels in live cells provided a quantitative analysis of the complex interrelationships between both processes. Our study shows that p53 activation differs even between cells that have a similar number of DNA breaks. Understanding the origin and consequences of such variability in normal and cancerous cells is crucial for developing efficient and selective therapeutic interventions.

## Background

The tumor suppressor p53 mediates the cellular response to DNA damage by triggering cell cycle arrest and DNA repair or by evoking cellular senescence and apoptosis [1]. These functions of p53 are essential for preserving genomic integrity and preventing neoplastic transformation. Loss of p53 activity, either by functional inactivation of its pathway or by gene mutation, is a frequent event in the onset and progression of many human malignancies [2,3]. p53 function is also critical to the efficacy of cancer therapies that generate DNA damage, such as radiation and chemotherapy, and defects in p53 are often associated with therapy resistance [1,4].

Within cells, levels of p53 protein are tightly controlled by several regulatory feedback loops that direct its stability and degradation. One major regulator of p53 is the E3 ubiquitin ligase Mdm2. p53 transcriptionally activates Mdm2 expression and Mdm2 targets p53 for degradation by the proteasome [5-8]. This interaction keeps p53 levels low under unstressed conditions. In response to cellular stress, p53 is activated through upstream kinases that induce post-translational modifications and disrupt the p53-Mdm2 interaction, allowing p53 to accumulate in the nucleus and induce the expression of target genes that mediate the cellular stress response [9].

DNA double strand breaks (DSBs) are particularly dangerous lesions for metazoan cells, as they can promote tumorigenesis by inducing chromosomal translocations and genomic instability upon misrepair [10]. A complex molecular machinery recognizes the presence of free DNA ends and induces the rapid activation of the kinase ataxia-telangiectasia mutated (ATM) [11-13] (reviewed in [14]). Among the substrates of ATM is the histone variant H2AX, which is phosphorylated around the break site and serves as a binding platform for mediator proteins that propagate the DNA damage signal.

Activated ATM subsequently phosphorylates and stabilizes p53, which shows a series of highly regulated, undamped pulses in single cells upon induction of DSBs. The amplitude and duration of p53 pulses is independent of the damage dose, whereas the number of pulses increases with higher damage [15]. These dynamics are a consequence of the feedback architecture of the p53 network. In addition to the p53-Mdm2 feedback, a second feedback mediated by the phosphatase Wip1 leads to periodic inactivation of ATM after a pulse of p53 accumulation [16]. This allows cells to evaluate the state of their genome and re-initiate the response if damage persists [17]. Moreover, p53 pulses after DSB induction are excitable: a complete p53 pulse of uniform amplitude and duration is induced independent of the strength of the damage signal [18,19]. Several theoretical studies have suggested potential physiological roles for p53 pulses [20-22]. Recently we have shown that, indeed, the dynamical behavior of p53 carries information that controls cell fate. Cells that experience p53 pulses undergo temporary cell cycle arrest and recover from the damage, while cells in which p53 shows a non-oscillatory, sustained response undergo apoptosis or senescence [23].

Although much insight has been gained into the molecular mechanisms that regulate p53 pulses in response to DSBs [16] and their functional role [23], little is known about the quantitative relationship of DNA damage and p53 induction. Specifically, is there a fixed threshold of damage that is necessary for activating a p53 response? Western blot analysis of ATM phosphorylation in irradiated cells suggested that ATM is activated in a highly sensitive manner. Damage doses estimated to generate one or two DSBs were sufficient for a partial activation of ATM, and doses that generated more than twenty DSBs evoked a complete ATM response [12,24]. Similarly, it was shown that cells are released from an ATM-mediated G2 checkpoint when less than approximately 20 DSBs remain [25]. The sensitivity of the p53 pathway was measured by introducing serial dilutions of restriction enzymes or linearized double-stranded DNA molecules into cells that were subsequently assayed for p53 function. From these studies, it was estimated that a solitary DSB might suffice to activate a p53 response [26].

However, measurements in previous studies averaged over populations of cells or estimated dynamics from fixed cells. Remarkably, identical cells in a uniformly damaged population exhibit a large heterogeneity in their p53 response, with cells showing a variable number of pulses [15,27]. It is conceivable that this variation arises from differences in the cells’ number of breaks or rates of repair. To gain a quantitative understanding of the relationship between the number of DSBs and p53 activation and to investigate the cause of heterogeneity in the p53 response, we established a cellular system that expresses fluorescent reporters to quantify both DNA damage and p53 dynamics in the same living cell.

## Results

### Quantification of DSBs and their rate of repair in individual living cells

**Figure 1:**
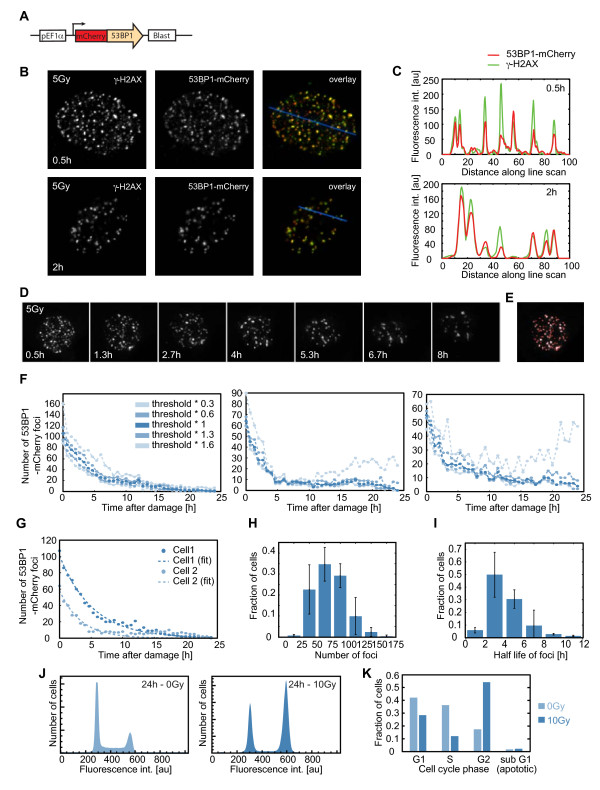
**Experimental system for quantifying DSBs in single, living cells. (A)** Schematic drawing of the 53BP1 reporter. **(B-C)** Cells expressing 53BP1-mCherry were fixed and stained with anti γ-H2AX antibody 30 minutes and 2 hours after 5Gy γ-irradiation. The overlaid image, and the measured intensities of both 53BP1-mCherry and γ-H2AX staining across a line in the nucleus (C) show co-localization between 53BP1 and γ-H2AX foci. **(D)** Time-lapse images of a cell expressing 53BP1-mCherry after 5Gy γ-irradiation. Images are maximum projections of z-stacks through the nucleus (see Methods section) in the mCherry channel. **(E)** Example of the automated segmentation for the enumeration of 53BP1-mCherry foci in a cell. Segmented foci are indicated as red circles. Image processing was performed using custom written Matlab based software (see Methods section for algorithmic details). **(F)** Enumerated 53BP1-mCherry foci for three cells using five different thresholds for foci detection. Except for very low levels, the quantification of foci is robust to changes in the threshold. **(G)** Enumerated 53BP1-mCherry foci (dots) and exponential fits to the raw data (dashed lines) for two cells. **(H-I)** Distribution of the initial number (H) and half-life (I) of 53BP1 foci in a population of cells treated with 5Gy γ-irradiation. The analysis was performed using a range of 0.6 to 1.3 times the optimal threshold level. Error bars indicate the standard deviation of the analyses performed at different threshold levels. Number of cells = 97. **(J-K)** Cell cycle distributions of untreated cells (0Gy) and irradiated cells (10Gy) showing a strong cell cycle arrest post irradiation with minimal death (sub G1 fraction). DSBs double strand breaks.

To quantify DNA DSBs in single cells, we used a fluorescent reporter system based on the mediator protein 53BP1 (Figure 1A). 53BP1 localizes to chromatin regions adjacent to DSBs within minutes after damage and forms foci that are discernable by light microscopy. These foci can serve as markers for the number and location of DSBs [28-32]. We established a clonal human MCF7 breast carcinoma cell line that stably expresses mouse 53BP1 fused to the fluorescent protein mCherry and verified that 53BP1 foci co-localize with the canonical marker for DSBs, γ-H2AX (Figure 1B, C and [32]). To follow the dynamics of DSBs over time, we performed live-cell time-lapse microscopy of reporter cells after treatment with ionizing radiation (Figure 1D). Using optical sectioning, deconvolution and automated image analysis, we quantified the number of 53BP1 foci at regular intervals (40 minutes) for a period of 24 hours post-irradiation (Figure 1E, F, see Methods section for details). Our analysis showed that the number of 53BP1-mCherry foci in a cell decreases with time. The decay in the number of foci was fitted to an exponential model (Figure 1G) and the half-life of 53BP1 foci was calculated for each cell [32-34]. We found that individual cells in a uniformly irradiated population acquire different initial numbers of 53BP1 foci (Figure 1H) and vary in their kinetics of repair (Figure 1I). Note that the main cellular outcome following DSBs is G1 and G2 arrest and not apoptosis, even in response to high levels of damage (Figure 1J, K and [23]).

**Figure 2:**
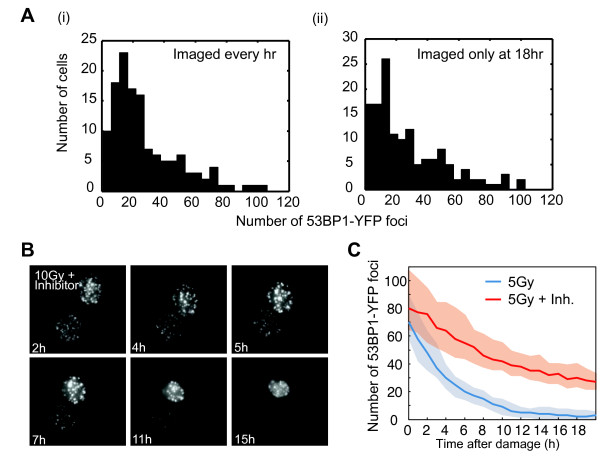
**Validation of the DSB reporter cell line. (A)** Distributions of the number of 53BP1-mCherry foci at 18 hours post damage in cells imaged every hour for 18 hours (i) or imaged only once at 18 hours (ii) post damage. Number of cells = approximately 140 for each condition; *P*-value 0.41, Kolmogorov-Smirnov test. **(B)** Time-lapse images of two cells that were pre-treated with a small molecule inhibitor of DNA-PK (NU7026) prior to 5Gy γ-irradiation. **(C)** Number of DSBs over time in cells treated with 5Gy γ-irradiation alone (blue) or in the presence of DNA-PK inhibitor (red). Solid lines represent the median, shaded areas the 25^th^ to 75^th^ percentile. Number of cells = 97 (5Gy) and n = 81 (5Gy + inhibitor). DSB, double strand break.

To validate that the decay in the number of foci represents repair (and not loss of signal due to photobleaching), we confirmed that the distribution of the number of foci at 18 hours post irradiation is similar between cells that were imaged frequently (every hour) and cells that were imaged only at 18 hours post irradiation (Figure 2A, *P*-value 0.41, Kolmogorov-Smirnov test). In addition, we treated cells with a specific small molecule inhibitor of DNA-PK (NU7026) [35]. This abrogated DNA repair by non-homologous end joining and led to a slower disappearance of foci, as DNA damage can be repaired only by homologous recombination in the presence of this drug (Figure 2B, C). Taken together, our results show a large heterogeneity in the induction and repair of DNA damage in identical cells exposed to the same damage dose.

### Determining the quantitative relationship between DSBs and activation of p53

**Figure 3:**
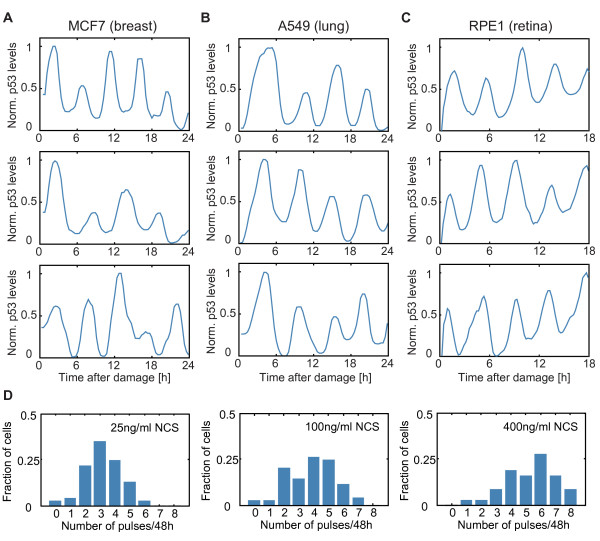
**Human cell lines show a series of p53 pulses in response to DSBs. (A-C)** The p53-Venus reporter was expressed in three lines: MCF7 - breast cancer **(A)**; A549 - lung cancer **(B)**; and RPE1 - retinal epithelial, non-cancerous **(C)**. Shown are representative examples of p53 trajectories in individual cells following DSBs (10Gy γ-irradiation). **(D)** MCF7 cells expressing the p53-Venus reporter were treated with the indicated doses of neocarcinostatin (NCS) and the number of p53 pulses post-damage was quantified. In each condition, the p53 response shows a high degree of heterogeneity. Number of cells >100 for each condition. DSBs, double strand breaks.

Induction of DNA damage leads to activation of the p53 network. To quantify the dynamics of p53 accumulation in single cells, we used a fluorescent reporter of p53 (p53-Venus). In previous studies, we have shown that the p53-Venus fusion protein faithfully reports the dynamics of endogenous p53 in MCF7 cells [16,18]: high doses of ionizing radiation induce a series of uniform p53 pulses (Figure 3A). MCF7 cells harbor an amplification of the *PPM1D/Wip1* gene locus and express relatively high levels of the phosphatase Wip1, potentially affecting p53 dynamics [36,37]. To ensure that p53 pulses are not limited to cells with high levels of Wip1, we established our fluorescent p53 reporter system in A549 lung cancer cells and immortalized non-cancerous RPE1 cells and followed p53 dynamics post-damage (Figure 3B, C). In both cell lines, we detected p53 pulses similar to MCF7 cells. Moreover, p53 pulses have been previously reported in additional cell lines and *in vivo* using a p53 reporter in mice [38-40], suggesting that p53 pulses are not limited to the MCF7 cancer line, but represent a general cellular response to DSBs.

Our quantification of DSBs in individual cells showed a large heterogeneity in the induction and rate of repair between cells exposed to the same damage dose (Figure 1). Is there a comparable heterogeneity in the p53 response? To test this, we treated cells with varying doses of the radiomimetic drug neocarcinostatin (NCS) and quantified the number of p53 pulses. As previously reported, higher levels of damage led on average to higher numbers of p53 pulses. However, even at high damage doses, cells showed a large variability in the p53 response (Figure 3D and [15,18]).

**Figure 4:**
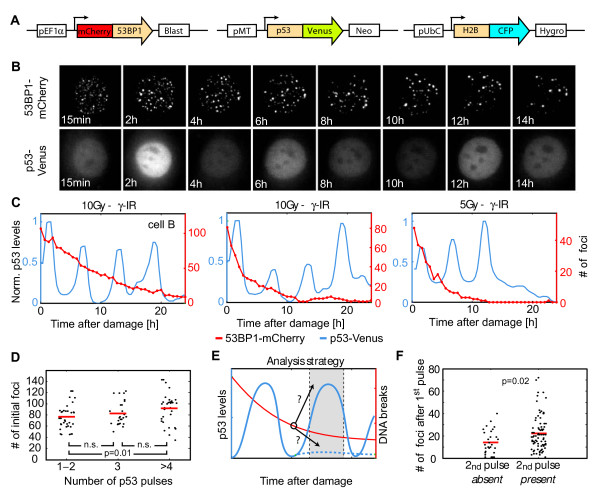
**Quantifying DSBs and p53 dynamics in individual living cells. (A)** Schematic drawing of the 53BP1, p53 and H2B reporters. **(B)** Time-lapse images of MCF7 cells expressing the reporters in **(A)** after damage (10Gy γ-irradiation) at the indicated time points. The 53BP1-mCherry images are maximum projections of z-stacks through the nucleus (see Methods section). **(C)** Quantification of the number of 53BP1-mCherry foci (red) and the normalized average nuclear p53-Venus intensity (blue). Trajectories from three individual cells treated with 5Gy or 10Gy γ-irradiation as indicated are shown. The left panel corresponds to the cell shown in **(B)**. **(D)** The correlation between the initial numbers of breaks post 5Gy and the number of p53 pulses during the response was analyzed. The median number of DSBs is significantly higher in cells that pulse four times or more compared to cells pulsing only once or twice, but the distributions are overlapping. Red lines indicate the median; *p*-values were calculated by the Wilcoxon-Mann–Whitney test. Number of cells = 97 **(E-F)** The correlation of DNA damage after a p53 pulse and the induction of a subsequent pulse was analyzed as indicated **(E)**. The number of breaks after the first p53 pulse is shown for individual cells, binned for the presence or absence of a subsequent pulse **(F)**. The medians are significantly different, but distributions overlap. For the analysis, data sets from cells treated with 5Gy or 10Gy γ-irradiation were combined. Red lines indicate the median; the *p*-value was calculated by the Wilcoxon-Mann–Whitney test. Number of cells >160. DSBs, double strand breaks.

We, therefore, asked whether the variability in the p53 response can be explained by the heterogeneity in the induction and repair of DBSs. To quantify the relationship between p53 pulses and DSBs we added the p53-Venus reporter to cells expressing the 53BP1-mCherry reporter (Figure 4A). We also added a fluorescent reporter for histone H2B (H2B-CFP) for obtaining a uniform nuclear signal that can aid with the automated segmentation of nuclei. We then treated cells with ionizing radiation and quantified the dynamics of DSB repair and p53 accumulation in individual cells over a time period of 24 hours (Figure 4B). We found that all cells show active repair. However, many cells still had residual breaks even 24 hours after irradiation. As expected, these cells show a continuous series of p53 pulses (Figure 4C, left panel). We also observed cells that apparently repaired all damage by 24 hours post irradiation. Surprisingly, these cells showed a heterogeneous p53 response: some cells continued to show p53 pulses (Figure 4C, middle panel), while in others, p53 returned to its basal level once repair was complete (Figure 4C, right panel). The variability in the number of p53 pulses was only poorly correlated with the initial number of breaks post damage (Figure 4D).

To analyze in more detail the relationship between DNA damage and the induction of a new p53 pulse during the repair process, we quantified the number of DSBs after a p53 pulse in each individual cell and correlated it with the presence or absence of a subsequent pulse in the expected time frame (Figure 4E). We found that cells showing a subsequent p53 pulse tended to have higher levels of DNA damage (Figure 4F). However, the distributions of retained damage between cells that showed a subsequent p53 pulse and cells that did not were broadly overlapping, and we were unable to observe a fixed threshold number of DSBs that determine whether p53 will pulse or not.

**Figure 5:**
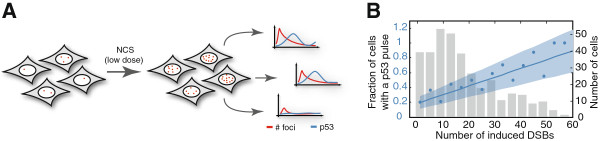
**The number of DNA double strand breaks determines the probability of a p53 pulse. (A)** Schematic representation of the experimental approach. Cells treated with low doses of NCS (0 to 100 ng/ml) were imaged for 8hr. Induced DSBs and p53 dynamics were quantified. **(B)** On the resulting data, we performed a robust linear regression (blue line) and calculated the corresponding confidence interval (α = 0.05, shaded blue area). For visualization, cells were binned according to the number of induced DSBs (bin size W = 4 foci) and the fraction of cells inducing a p53 pulse was plotted for each bin (blue dots). The number of cells in each bin is indicated by grey bars (total number of cells = approximately 350). DSBs, couble strand breaks; NCS, neocarcinostatin.

As we were unable to determine a fixed threshold of DSBs for the induction of p53 pulses during repair, we used an alternative approach: we generated a distribution of induced DSBs by treating cells with a range of low NCS doses and correlated the amount of damage to the induction of a p53 response (Figure 5A). Using NCS instead of ionizing radiation allowed us to treat cells directly on the microscope and quantify DSBs before and immediately after damage without a significant time delay in image acquisition. Moreover, we were able to finely titrate the amount of damaging agent to preferentially generate low numbers of DSBs, close to the previously suggested threshold levels [25,26]. We have previously shown that the kinetics of DSB repair following NCS treatment are similar to those observed after γ-irradiation [32].

To analyze the relationship between DNA breaks and the induction of p53, we measured the number of DSBs and p53 pulses in more than 350 cells post DNA damage. Cells were binned according to the number of DSBs, and the fraction of cells that induced a p53 pulse in each bin was plotted (Figure 5B). We expected to see a clear distinction between non-responding and responding cells at a defined threshold level of DSBs. Surprisingly, what we observed instead was a linear relationship between DNA damage and the p53 response: with higher amounts of damage, the fraction of cells responding with a p53 pulse increased continuously. This observation suggested that the amount of DNA damage in each cell determines the probability of activating a p53 response. For intermediate levels of breaks the p53 response is heterogeneous between cells; for example, only about 50% of cells with 20 breaks show a p53 pulse.

### The decision to activate a p53 pulse depends on previous exposure to DNA damage and additional cell-intrinsic factors

**Figure 6:**
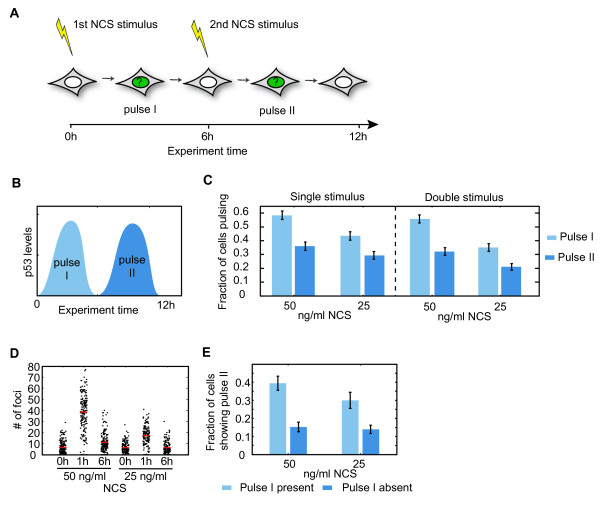
**Activation of p53 in response to repeated damage induction. (A)** Schematic representation of the experimental design. Cells were damaged with a low dose of NCS and analyzed for the activation of a p53 pulse. The same cells were re-damaged with the same dose of NCS 6hr after the first damage treatment. **(B)** Schematic representation of p53’s first pulse (pulse I; light blue) and second pulse (pulse II; dark blue). Note that since the duration of each p53 pulse is about five to six hours, the second NCS treatment was applied after the first pulse has been completed. **(C)** The fraction of cells showing pulse I (light blue) or pulse II (dark blue) in response to one NCS treatment (single stimulus) or two subsequent NCS treatments (double stimulus) at the indicated concentrations. The second treatment of NCS did not lead to a higher fraction of cells with pulse II. **(D)** The number of 53BP1 foci was quantified before and at the indicated time points after treatment with low doses of NCS (50 and 25 ng/ml). Red lines indicate the median. **(E)** The fraction of cells showing a p53 pulse in response to the second damage treatment is shown for two different NCS concentrations (light blue bars = cells that induced a p53 upon the first treatment, dark blue bars = cells that did not respond after the first stimulus). Error bars represent the standard error of the proportion. Number of cells = approximately 700. NCS, neocarcinostatin.

Previous single cell studies have shown that heterogeneity in cellular behavior can be based on different phenomena [41]; some cellular processes behave as stochastic systems based on the random fluctuations of their molecular components (for example, induction of apoptosis, [42]). Other processes are influenced by the cellular state, for example cell cycle phase [32]. To test whether the decision to activate a p53 pulse at intermediate amounts of DSBs is entirely stochastic, we treated cells with an initial low dose of damage and after six hours re-damaged them with the same damage dose (Figure 6A). We first compared the fraction of cells showing a p53 pulse (pulse I) in response to the first NCS treatment with the fraction of cells showing a pulse (pulse II) after the second NCS treatment (double stimulus). Surprisingly, we found fewer cells showing a pulse in response to the second treatment (Figure 6B, C). Moreover, the fraction of cells showing a pulse after the second treatment (double stimulus, pulse II) did not exceed the fraction of cells showing a second p53 pulse in response to only one treatment (single stimulus, pulse II), although the DNA damage was largely repaired at this point (Figure 6D). This suggests that during the first phase of the response (six hours) the p53 pathway does not reset and becomes desensitized to a second treatment. A similar behavior was recently reported for the activation of NFκB in response to repeated treatments of TNFα [43]. We next asked whether the cells that do show a pulse after the second treatment are also the ones that showed a pulse after the first treatment. Our analysis revealed that the probability of showing a second pulse was higher in cells that reacted upon the first stimulus (Figure 6E). Taken together our analysis shows that the generation of a p53 pulse in response to a distinct number of DSBs is not entirely stochastic; it is affected by previous exposure to stress and may be influenced by additional internal cell-specific factors.

### Which internal cellular factors may affect the decision to pulse or not?

**Figure 7:**
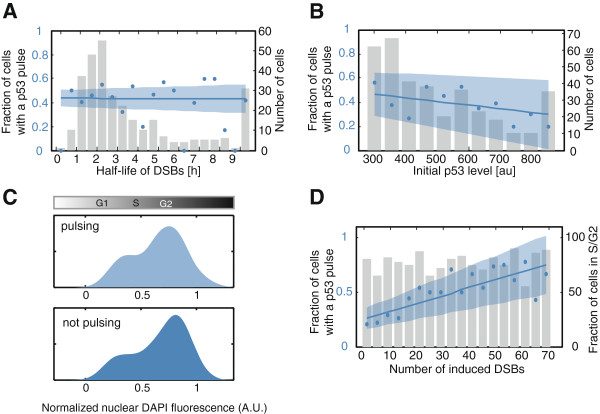
**The effect of cell-intrinsic factors on the p53 response. (A-B)** We correlated the half-life of 53BP1 foci **(A)** and basal p53-Venus levels prior to damage **(B)** with the presence or absence of a p53 pulse in individual cells (blue line: robust linear fit, shaded blue area: confidence interval at α = 0.05). For visualization, we plotted the fraction of cells inducing a p53 pulse after damage binned according to their half-life of 53BP1 foci (**A**, bin size W = two hours, blue dots) or basal p53-Venus level prior to damage (**B**, bin size W = 50 au, blue dots). The number of cells in each bin is indicated by grey bars. Number of cells = approximately 300. **(C)** Cell cycle distribution of cells that induce a p53 pulse (light blue) or do not pulse (dark blue) after damage (50 ng/ml NCS). *P*-value 0.6337, Kolmogorov-Smirnov test **(D)** The number of induced DSBs and the resulting p53 response are correlated (blue line: robust linear fit, shaded blue area: confidence interval at α = 0.05). The fraction of cells inducing a p53 pulse after damage is plotted for cells binned according to the number of induced 53BP1 foci (bin size W = four foci, blue dots). For each bin, the percentage of cells in S/G2 phases is indicated by grey bars. Cells were classified into G1 and S/G2 groups based on their normalized DAPI fluorescence (G1 cells: <0.33 normalized DAPI fluorescence units and S/G2 cells: > = 0.33 normalized DAPI fluorescence units). Number of cells = 270.

We tested three cellular processes that could potentially influence the sensitivity of the p53 response: rate of DNA repair, the level of p53 itself and the cell cycle phase. First, we tested if the induction of p53 pulses is influenced by the activity of the cellular DNA repair machinery, which is reflected in the kinetics of repair. Cells that achieve rapid recognition and repair of DSBs may not initiate a p53 pulse in response to damage, while cells that are slower in their response to DNA DSBs may activate p53 to induce cell-cycle arrest and allow additional time for repair. To test this hypothesis, we plotted the fraction of cells inducing a p53 pulse binned according to their half-lives of DSBs (Figure 7A). The lack of correlation between the rates of repair and the probability of activating p53 post damage indicates independence of the p53 response from the efficiency of the repair machinery.

We previously showed that p53 is frequently activated in proliferating cells [18]. As these spontaneous pulses of p53 activate negative feedback mechanisms, the sensitivity of the p53 network may depend on its previous activation state. Indeed, we saw here that previous exposure to DNA damage desensitizes the p53 response (Figure 6C). We, therefore, analyzed whether p53 absolute levels pre-damage influence the activation of p53 pulses post-damage. We found that cells with high initial p53 levels prior to NCS tended to have a lower probability of inducing a p53 pulse post-damage (Figure 7B). However, the limited correlation between both parameters indicates that basal p53 levels *per se* are not a good predictor for the subsequent p53 response. Note that since p53 initial levels were determined by measuring a single time point prior to NCS, cells with low levels of p53 might have just completed a p53 pulse and may still be in a desensitized state.

Finally, we tested if activation of p53 post-damage is determined by the cell cycle phase [44]. It is possible that cells in different cell cycle phases vary in their sensitivity to DNA damage and have distinct thresholds of DSBs necessary for activating p53. To investigate this, we imaged damaged cells to quantify the dynamics of their DSB repair and p53 activation. We then calculated the cell cycle phase of the imaged cells by measuring their DNA content using diamidino-2-phenylindole (DAPI) staining (see Methods section for details). We found that cells that induce a p53 pulse and cells that do not activate a p53 response had similar cell cycle distributions (*P*-value 0.6337, Kolmogorov-Smirnov test, Figure 7C). To explore if different cell cycle phases vary in the threshold number of DSBs required to induce p53, we binned cells according to their initial numbers of DSBs and plotted both the fraction of cells inducing a p53 pulse and the proportion of S/G2 cells for each bin (Figure 7D). We found a uniform proportion of S/G2 cells across all bins indicating that cells in different cell cycle phases do not differ in their thresholds for activating a p53 pulse.

## Discussion

Our data indicate a linear relationship between the amount of DSBs in a cell and the probability that p53 will pulse. However, even for a fixed number of breaks, some cells show a pulse and others do not. Such heterogeneity in the response of individual cells has been frequently observed in other biological systems. For example, in response to low doses of the tumor necrosis factor, TNFα, nuclear localization of the transcription factor NFκB is only observed in a few cells. With higher doses of TNFα, more and more cells respond to the input [45]. While the factors responsible for cell-cell variability were successfully identified for some biological systems [46,47], in many cases the origin of heterogeneous cellular responses remains elusive.

One potential cause of heterogeneity between cells in the cellular response is that cells receive varying amounts of input, for example, ligands or drugs. Here, we directly measure the input each cell receives by enumerating the number of DSB. However, we found large variations in the induction of p53 even between cells that have similar numbers of DSBs, suggesting that it is not the level of DSB per se that explains the cell-cell variability in the decision to activate a p53 pulse.

By looking at the same cell in response to two rounds of DNA damage we showed that the p53 pathway does not reset after the response to the first stimulus, even when most of the damage is repaired. This indicates that the decision to activate a p53 pulse is affected by previous exposure to damage. In addition, the probability to show a second pulse was higher in cells that also had a pulse in response to the first stimulus suggesting that the decision whether to activate p53 in response to low amounts of DSBs is not entirely stochastic, but is likely affected by the internal state of individual cells. Although our analysis of the three cellular processes most likely to affect the sensitivity of the p53 network (rate of DNA repair, cell cycle phase and initial levels of p53) did not reveal a major influence, there are other factors that may contribute to setting individual thresholds for p53 activation. Such factors might include the expression of key proteins that regulate p53, such as the negative regulator Mdm2. The stimulus provided by DSBs may not be sufficient to initiate a p53 pulse in cells that express high levels of Mdm2. Interestingly, it was recently reported that tumor growth factor β (TGFβ) signaling attenuates the p53-mediated stress response [48]. Other signaling pathways may interact with p53 as well. Using our experimental system, it would now be feasible to alter the signal state of cells systematically and determine the sensitivity of the p53 response.

Our analyses showed that some cells do not activate p53 even at high levels of DNA damage. One possibility for this observation is that the induction of p53 in response to DSBs is highly deregulated in cancer cells. It will be important to determine if normal, non-transformed cells are more uniform in their p53 response and show activation of p53 at a low number of DSBs. Similar investigations carried out in multiple tumor cell lines will enable an understanding of their potential to uniformly induce p53 in response to DNA damage and will provide insights into their sensitivity to radiation and chemotherapeutic treatments.

In this work we looked at the relationship between DSB and p53 induction, and the variation between cells, in an unperturbed system. One question that arises from our study is whether this relationship can be altered when DNA repair is inhibited. This is especially important as major pharmaceutical companies have begun significant projects attempting to inhibit specific proteins in DNA repair pathways, with the goal of using DNA repair inhibitors in combination with DNA-damaging treatments to prevent repair and trigger death or cell cycle arrest. The same question can be asked in the opposite direction – how does perturbation of p53 dynamics affect the rate of repair? Recent studies from our group and others have shown that the dynamical behavior of p53 encodes critical cell-fate decisions [23,39]; hence, understanding how perturbations of p53 and key repair proteins will provide new and important insights for the treatment of tumors with different genetic profiles and repair deficiencies.

## Conclusions

In this study we combined a reporter for DSBs with a fluorescent reporter for p53 and quantified the level of damage and the dynamics of p53 in the same, living cell. We found a linear correlation between the number of DSBs and the probability for activating a p53 pulse; more DSBs increase the probability that a cell will have a p53 pulse. However, there was no distinct threshold of damage for inducing a p53 response. By re-damaging cells we showed that the decision to activate p53 is not entirely stochastic but is determined by both previous exposure to DNA damage and additional internal cell-specific factors. However, cell cycle phase, the initial levels of p53 and the rate of repair are not major determinants of this decision. The use of fluorescent reporters to quantify DNA damage and p53 levels in live cells now presents powerful tools for providing an integrated quantitative understanding of their complex interrelationships in normal and cancerous cells.

## Methods

### Cell culture

Human breast cancer epithelial MCF7 and A549 cells were grown in RPMI 1640 medium supplemented with 10% fetal calf serum, 100 U/mL penicillin, 100 μg/mL streptomycin and 250 ng/mL fungizone (Gemini Bio-Products, West Sacramento, CA, USA). RPE1-hTERT cells were grown in (D)MEM/F12 medium supplemented with 10% fetal calf serum, penicillin, streptomycin and fungizone. When required, the medium was supplemented with selective antibiotics (400 μg/mL G418, 5 μg/mL blasticidin, 50 μg/mL hygromycin). When indicated, medium was replaced with fresh medium supplemented with neocarzinostatin (National Cancer Institute, Bethesda, MD, USA) or with the DNA-PK inhibitor NU7026 (used at 10 μM, Sigma-Aldrich, St. Louis, MO, USA) during experiments. Irradiation treatments were carried out in a ^60^Co irradiator. Cell cycle distributions were analyzed by DAPI staining.

### Cell line construction

The original pCMV-EGFP-53BP1 construct was kindly provided by Prof. Yasuhisa Adachi (Jullien *et al*. [30]). We generated our pEF1α-mCherry-53BP1 plasmid by replacing GFP with mCherry and combining this fluorescent protein-cDNA fragment with the EF1α promoter in a vector harboring a blasticidin resistance cassette using standard molecular biology techniques. This plasmid was stably transfected into MCF7 cells using FuGENE6 (Hoffmann-La Roche, USA), which were maintained in selective media and sorted into single cells using fluorescence activated cell sorting to generate a clonal population. Our pMT-p53-Venus plasmid has been previously reported [16]. Stable, clonal cell lines were established as described above.

For constructing the pUbC-H2B-CFP vector, the H2B coding sequence was amplified by PCR from the vector pBOS-H2BGFP (BD Bioscience, San Jose, CA, USA). Using Multiside Gateway technology (Invitrogen, Eugene, OR, USA), the PCR product was combined with the Ubiquitin C promoter and CFP tag in a lentiviral vector harboring a hygromycin resistance cassette. This plasmid was transfected into 293T cells together with the corresponding packaging plasmids to generate replication-defective viral particles using standard protocols, which were used to stably infect the engineered MCF7 cell line.

### Time-lapse microscopy

Cells were plated in RMPI lacking riboflavin and phenol red in poly-D-lysine coated glass-bottom plates (MatTek Corporation, Ashland, MA, USA) 24 hours prior to microscopy}. The medium was supplemented with 10% fetal calf serum, 100 U/mL penicillin, 100 μg/mL streptomycin, 250 ng/mL fungizone (Gemini Bio-Products) and 10 mM HEPES. Cells were imaged on a Nikon Eclipse Ti inverted microscope with a Plan Apo 60X oil objective (NA 1.4), Hamamatsu Orca ER camera and a Perfect Focus System. The microscope was surrounded by a custom enclosure to maintain constant temperature and atmosphere. The filter sets used were CFP: 436/20 nm; 455 nm; 480/40 nm (excitation; beam splitter; emission filter, respectively), YFP: 500/20 nm; 515 nm; 535/30 nm; and mCherry: 560/40 nm; 585 nm; 630/75 nm (Chroma, Bellows Falls, VT, USA). Images were acquired every 15 to 20 minutes in the phase, YFP and CFP channels and every 15 to 40 minutes in the mCherry channel for 8 to 12 hours. We acquired seven z-sections with a step size of 1 μm in the mCherry channel. Image acquisition was controlled by MetaMorph software (Molecular Devices, Sunnyvale, CA, USA).

For analyzing cell cycle distribution, cells were imaged for six hours post-damage as described above, fixed with 2% paraformaldehyde, permeabilized with 0.2% Triton/PBS and stained with Hoechst (Molecular Probes, Eugene, OR, USA). We imaged thousands of cells and quantified the integrated fluorescence intensity of the Hoechst signal by image analysis using automated thresholding and watershed algorithms to segment individual nuclei. Using the nuclear intensity of the DNA dye, we established a histogram of the distribution of DNA content that allowed assigning a cell cycle phase to each cell. We identified cells analyzed in the preceding time-lapse experiment using gridded cover slips.

### Image analysis

Custom written algorithms in Matlab (Mathworks) were used to analyze 53BP1 foci. In brief, image stacks were first enhanced using blind deconvolution (AutoQuant) and were then converted to two-dimensional maximum projections. Nuclei were segmented using the H2B-CFP signal. For each nucleus, the background signal was first reduced by a Tophat transformation, following which the edges were detected using the Canny method. Foci were determined from the edges using morphological transformations and optimal thresholding. To determine the effect of thresholding on our foci measurements, we increased and decreased the threshold by a factor of up to 0.3 and 1.6, respectively, and determined the effect on foci quantification. Touching foci were separated by a marker-directed watershed algorithm. We analyzed p53 trajectories in single cells using previously described algorithms [18]. The raw data are available on request.

### Immunofluorescence

Cells were grown on number 1.5 glass coverslips coated with poly-L-lysine (Sigma-Aldrich, St. Louis, MO, USA). They were fixed with 2% paraformaldehyde, permeabilized with 0.2% Triton/PBS and blocked with 5% goat serum supplemented with 1% bovine serum albumin. Cells were treated with primary antibody to detect γ-H2AX (mouse monoclonal JBW301, Upstate Millipore, Billerica, MA, USA, 1:700 dilution), washed and treated with secondary antibody conjugated with Alexa Fluor 647 (Molecular Probes). After washing, cells were stained with Hoechst (Molecular Probes) and embedded in Prolong Antifade (Invitrogen). Immunofluorescence preparations were imaged on the microscope described for live cell imaging and automated segmentation was performed in Matlab (MathWorks) with algorithms from CellProfiler [49].

## Competing interests

The authors declare that they have no competing interests.

## Authors’ contribution

AL and GL designed the experiments, AL and KK performed the experiments and analyzed the data, CM established A549 cells expressing the p53 reporter and acquired the single cell data. AL, KK and GL wrote the manuscript. All authors read and approved the final manuscript.

## References

[B1] Vousden KH Lane DP p53 in health and disease Nat Rev Mol Cell Biol 2007 11 275 283 10.1038/nrm2147 17380161

[B2] Muller PAJ Vousden KH p53 mutations in cancer Oncogene 2013 11 2 8 10.1038/ncb2641 23263379

[B3] Brosh R Rotter V When mutants gain new powers: news from the mutant p53 field Nat Rev Cancer 2009 11 701 713 19693097 10.1038/nrc2693

[B4] Jiang H Reinhardt H Bartkova J Tommiska J Blomqvist C Nevanlinna H Bartek J Yaffe M Hemann M The combined status of ATM and p53 link tumor development with therapeutic response Genes Dev 2009 11 1895 1909 10.1101/gad.1815309 19608766 PMC2725944

[B5] Barak Y Juven T Haffner R Oren M mdm2 expression is induced by wild type p53 activity EMBO J 1993 11 461 468 8440237 10.1002/j.1460-2075.1993.tb05678.x PMC413229

[B6] Haupt Y Maya R Kazaz A Oren M Mdm2 promotes the rapid degradation of p53 Nature 1997 11 296 299 10.1038/387296a0 9153395

[B7] Kubbutat MH Jones SN Vousden KH Regulation of p53 stability by Mdm2 Nature 1997 11 299 303 10.1038/387299a0 9153396

[B8] Wu X Bayle JH Olson D Levine AJ The p53-mdm-2 autoregulatory feedback loop Genes Dev 1993 11 1126 1132 10.1101/gad.7.7a.1126 8319905

[B9] Kruse J Gu W Modes of p53 regulation Cell 2009 11 609 622 10.1016/j.cell.2009.04.050 19450511 PMC3737742

[B10] Hoeijmakers JH Genome maintenance mechanisms for preventing cancer Nature 2001 11 366 374 10.1038/35077232 11357144

[B11] Ahn JY Schwarz JK Piwnica-Worms H Canman CE Threonine 68 phosphorylation by ataxia telangiectasia mutated is required for efficient activation of Chk2 in response to ionizing radiation Cancer Res 2000 11 5934 5936 11085506

[B12] Bakkenist CJ Kastan MB DNA damage activates ATM through intermolecular autophosphorylation and dimer dissociation Nature 2003 11 499 506 10.1038/nature01368 12556884

[B13] Matsuoka S Rotman G Ogawa A Shiloh Y Tamai K Elledge SJ Ataxia telangiectasia-mutated phosphorylates Chk2 in vivo and in vitro Proc Natl Acad Sci U S A 2000 11 10389 10394 10.1073/pnas.190030497 10973490 PMC27034

[B14] Ciccia A Elledge SJ The DNA damage response: making it safe to play with knives Mol Cell 2010 11 179 204 10.1016/j.molcel.2010.09.019 20965415 PMC2988877

[B15] Lahav G Rosenfeld N Sigal A Geva-Zatorsky N Levine AJ Elowitz MB Alon U Dynamics of the p53-Mdm2 feedback loop in individual cells Nat Genet 2004 11 147 150 10.1038/ng1293 14730303

[B16] Batchelor E Mock CS Bhan I Loewer A Lahav G Recurrent initiation: a mechanism for triggering p53 pulses in response to DNA damage Mol Cell 2008 11 277 289 10.1016/j.molcel.2008.03.016 18471974 PMC2579769

[B17] Batchelor E Loewer A Lahav G The ups and downs of p53: understanding protein dynamics in single cells Nat Rev Cancer 2009 11 371 377 10.1038/nrc2604 19360021 PMC2892289

[B18] Loewer A Batchelor E Gaglia G Lahav G Basal dynamics of p53 reveal transcriptionally attenuated pulses in cycling cells Cell 2010 11 89 100 10.1016/j.cell.2010.05.031 20598361 PMC3003696

[B19] Batchelor E Loewer A Mock C Lahav G Stimulus-dependent dynamics of p53 in single cells Mol Syst Biol 2011 11 488 21556066 10.1038/msb.2011.20 PMC3130553

[B20] Mengel B Hunziker A Pedersen L Trusina A Jensen MH Krishna S Modeling oscillatory control in NF-κB, p53 and Wnt signaling Curr Opin Genet Dev 2010 11 656 664 10.1016/j.gde.2010.08.008 20934871

[B21] Sun T Chen C Wu Y Zhang S Cui J Shen P Modeling the role of p53 pulses in DNA damage- induced cell death decision BMC Bioinformatics 2009 11 190 10.1186/1471-2105-10-190 19545411 PMC2713228

[B22] Ma L A plausible model for the digital response of p53 to DNA damage Proc Natl Acad Sci U S A 2005 11 14266 14271 10.1073/pnas.0501352102 16186499 PMC1242279

[B23] Purvis JE Karhohs KW Mock C Batchelor E Loewer A Lahav G p53 dynamics control cell fate Science 2012 11 1440 1444 10.1126/science.1218351 22700930 PMC4162876

[B24] Buscemi G Perego P Carenini N Nakanishi M Chessa L Chen J Khanna K Delia D Activation of ATM and Chk2 kinases in relation to the amount of DNA strand breaks Oncogene 2004 11 7691 7700 10.1038/sj.onc.1207986 15361830

[B25] Deckbar D Birraux J Krempler A Tchouandong L Beucher A Walker S Stiff T Jeggo P Löbrich M Chromosome breakage after G2 checkpoint release J Cell Biol 2007 11 749 755 10.1083/jcb.200612047 17353355 PMC2064048

[B26] Huang LC Clarkin KC Wahl GM Sensitivity and selectivity of the DNA damage sensor responsible for activating p53-dependent G1 arrest Proc Natl Acad Sci U S A 1996 11 4827 4832 10.1073/pnas.93.10.4827 8643488 PMC39364

[B27] Geva-Zatorsky N Rosenfeld N Itzkovitz S Milo R Sigal A Dekel E Yarnitzky T Liron Y Polak P Lahav G Alon U Oscillations and variability in the p53 system Mol Syst Biol 2006 11 2006.0033 16773083 10.1038/msb4100068 PMC1681500

[B28] Anderson L Henderson C Adachi Y Phosphorylation and rapid relocalization of 53BP1 to nuclear foci upon DNA damage Mol Cell Biol 2001 11 1719 1729 10.1128/MCB.21.5.1719-1729.2001 11238909 PMC86718

[B29] Bekker-Jensen S Lukas C Melander F Bartek J Lukas J Dynamic assembly and sustained retention of 53BP1 at the sites of DNA damage are controlled by Mdc1/NFBD1 J Cell Biol 2005 11 201 211 10.1083/jcb.200503043 16009723 PMC2171401

[B30] Jullien D Vagnarelli P Earnshaw WC Adachi Y Kinetochore localisation of the DNA damage response component 53BP1 during mitosis J Cell Sci 2002 11 71 79 11801725 10.1242/jcs.115.1.71

[B31] Schultz LB Chehab NH Malikzay A Halazonetis TD p53 binding protein 1 (53BP1) is an early participant in the cellular response to DNA double-strand breaks J Cell Biol 2000 11 1381 1390 10.1083/jcb.151.7.1381 11134068 PMC2150674

[B32] Karanam K Kafri R Loewer A Lahav G Quantitative live cell imaging reveals a gradual shift between DNA repair mechanisms and a maximal use of HR in mid S phase Mol Cell 2012 11 320 329 10.1016/j.molcel.2012.05.052 22841003 PMC3494418

[B33] Noon AT Shibata A Rief N Löbrich M Stewart GS Jeggo PA Goodarzi AA 53BP1-dependent robust localized KAP-1 phosphorylation is essential for heterochromatic DNA double-strand break repair Oncogene 2010 11 177 184 10.1038/ncb2017 20081839

[B34] Shibata A Conrad S Birraux J Geuting V Barton O Ismail A Kakarougkas A Meek K Taucher-Scholz G Löbrich M Jeggo PA Factors determining DNA double-strand break repair pathway choice in G2 phase EMBO J 2011 11 1079 1092 10.1038/emboj.2011.27 21317870 PMC3061033

[B35] Veuger SJ Curtin NJ Richardson CJ Smith GCM Durkacz BW Radiosensitization and DNA repair inhibition by the combined use of novel inhibitors of DNA-dependent protein kinase and poly(ADP-ribose) polymerase-1 Cancer Res 2003 11 6008 6015 14522929

[B36] Bulavin DV Demidov ON Saito S Kauraniemi P Phillips C Amundson SA Ambrosino C Sauter G Nebreda ÁR Anderson CW Kallioniemi A Fornace AJ Appella E Amplification of PPM1D in human tumors abrogates p53 tumor-suppressor activity Nat Genet 2002 11 210 215 10.1038/ng894 12021785

[B37] Li J Yang Y Peng Y Austin RJ van Eyndhoven WG Nguyen KCQ Gabriele T McCurrach ME Marks JR Hoey T Lowe SW Powers S Oncogenic properties of PPM1D located within a breast cancer amplification epicenter at 17q23 Nat Genet 2002 11 133 134 10.1038/ng888 12021784

[B38] Hamstra DA Bhojani MS Griffin LB Laxman B Ross BD Rehemtulla A Real-time evaluation of p53 oscillatory behavior in vivo using bioluminescent imaging Cancer Res 2006 11 7482 7489 10.1158/0008-5472.CAN-06-1405 16885345

[B39] Chen X Chen J Gan S Guan H Zhou Y Ouyang Q Shi J DNA damage strength modulates a bimodal switch of p53 dynamics for cell-fate control BMC Biol 2013 11 73 10.1186/1741-7007-11-73 23800173 PMC3702437

[B40] Hu W Feng Z Ma L Wagner J Rice JJ Stolovitzky G Levine AJ A single nucleotide polymorphism in the MDM2 gene disrupts the oscillation of p53 and MDM2 levels in cells Cancer Res 2007 11 2757 2765 10.1158/0008-5472.CAN-06-2656 17363597

[B41] Loewer A Lahav G We are all individuals: causes and consequences of non-genetic heterogeneity in mammalian cells Curr Opin Genet Dev 2011 11 753 758 22005655 10.1016/j.gde.2011.09.010 PMC3270938

[B42] Spencer SL Gaudet S Albeck JG Burke JM Sorger PK Non-genetic origins of cell-to-cell variability in TRAIL-induced apoptosis Nature 2009 11 428 432 10.1038/nature08012 19363473 PMC2858974

[B43] Ashall L Horton CA Nelson DE Paszek P Harper CV Sillitoe K Ryan S Spiller DG Unitt JF Broomhead DS Kell DB Rand DA Sée V White MRH Pulsatile stimulation determines timing and specificity of NF-kappaB-dependent transcription Science 2009 11 242 246 10.1126/science.1164860 19359585 PMC2785900

[B44] Beucher A Birraux J Tchouandong L Barton O Shibata A Conrad S Goodarzi A Krempler A Jeggo P Löbrich M ATM and Artemis promote homologous recombination of radiation-induced DNA double-strand breaks in G2 EMBO J 2009 11 3413 3427 10.1038/emboj.2009.276 19779458 PMC2752027

[B45] Tay S Hughey JJ Lee TK Lipniacki T Quake SR Covert MW Single-cell NF-κB dynamics reveal digital activation and analogue information processing Nature 2010 11 267 271 10.1038/nature09145 20581820 PMC3105528

[B46] Cohen AA Geva-Zatorsky N Eden E Frenkel-Morgenstern M Issaeva I Sigal A Milo R Cohen-Saidon C Liron Y Kam Z Cohen L Danon T Perzov N Alon U Dynamic proteomics of individual cancer cells in response to a drug Science 2008 11 1511 1516 10.1126/science.1160165 19023046

[B47] Snijder B Pelkmans L Origins of regulated cell-to-cell variability Nat Rev Mol Cell Biol 2011 11 119 125 10.1038/nrm3044 21224886

[B48] López-Díaz FJ Gascard P Balakrishnan SK Zhao J del Rincon SV Spruck C Tlsty TD Emerson BM Coordinate transcriptional and translational repression of p53 by TGF-β1 impairs the stress response Mol Cell 2013 11 552 564 10.1016/j.molcel.2013.04.029 23706820 PMC3735454

[B49] Carpenter AE Jones TR Lamprecht MR Clarke C Kang IH Friman O Guertin DA Chang JH Lindquist RA Moffat J Golland P Sabatini DM Cell Profiler: image analysis software for identifying and quantifying cell phenotypes Genome Biol 2006 11 R100 10.1186/gb-2006-7-10-r100 17076895 PMC1794559

